# Serum depletion induced cancer stem cell-like phenotype due to nitric oxide synthesis in oncogenic *HRas* transformed cells

**DOI:** 10.18632/oncotarget.12117

**Published:** 2016-09-19

**Authors:** Keisuke Monji, Takeshi Uchiumi, Saki Hoshizawa, Mikako Yagi, Takashi Matsumoto, Daiki Setoyama, Yuichi Matsushima, Kazuhito Gotoh, Rie Amamoto, Donchon Kang

**Affiliations:** ^1^ Department of Clinical Chemistry and Laboratory Medicine, Graduate School of Medical Sciences, Kyushu University, Higashi-ku, Fukuoka 812-8582, Japan; ^2^ Department of Nutritional Sciences, Faculty of Health and Welfare, Seinan Jo Gakuin University, Kokurakita-ku, Kitakyushu 803-0835, Japan

**Keywords:** serum depletion, nitric oxide, cancer stem cell, HRas, OXPHOS

## Abstract

Cancer cells rewire their metabolism and mitochondrial oxidative phosphorylation (OXPHOS) to promote proliferation and maintenance. Cancer cells use multiple adaptive mechanisms in response to a hypo-nutrient environment. However, little is known about how cancer mitochondria are involved in the ability of these cells to adapt to a hypo-nutrient environment. Oncogenic *HRas* leads to suppression of the mitochondrial oxygen consumption rate (OCR), but oxygen consumption is essential for tumorigenesis. We found that in oncogenic *HRas* transformed cells, serum depletion reversibly increased the OCR and membrane potential. Serum depletion promoted a cancer stem cell (CSC)-like phenotype, indicated by an increase in CSC markers expression and resistance to anticancer agents. We also found that nitric oxide (NO) synthesis was significantly induced after serum depletion and that NO donors modified the OCR. An NOS inhibitor, SEITU, inhibited the OCR and CSC gene expression. It also reduced anchorage-independent growth by promoting apoptosis. In summary, our data provide new molecular findings that serum depletion induces NO synthesis and promotes mitochondrial OXPHOS, leading to tumor progression and a CSC phenotype. These results suggest that mitochondrial OCR inhibitors can be used as therapy against CSC.

## INTRODUCTION

Mitochondria are responsible for the generation of adenosine triphosphate (ATP) through oxidative phosphorylation (OXPHOS) and they also play vital roles in β-oxidation, Ca^2+^ buffering, apoptosis, and reactive oxygen species (ROS) production [1, 2]. In tumors and other proliferating cells, the rate of glucose uptake and lactate production dramatically increases, even in the presence of oxygen. Otto Warburg's theory suggests that tumor cells have defects in mitochondrial OXPHOS and therefore rely on high levels of aerobic glycolysis as a major source of ATP to fuel cellular proliferation (the Warburg effect) [3]. While mitochondria are functional in tumor cells, many argue that decreases in mitochondrial metabolism and respiratory rate are essential for tumor cell proliferation [4]. Conversely, several studies have demonstrated the importance of mitochondria in driving the process of malignant cell transformation [5, 6].

It is becoming increasingly evident that a particular sub-population of tumor cells plays a critical role in tumorigenesis. This subpopulation is commonly known as tumor-initiating cells, or cancer stem cells (CSCs), and they have been identified in many types of cancer. Aberrant expression of *Oct4*, *Nanog*, *Sox2* and *Klf4* are associated with abnormal tissue growth and tumorigenesis [7–9]. These CSCs are defined by two key characteristics, enhanced tumorigenicity and the capacity for self-renewal/differentiation [10, 11]. CSCs are also relatively resistant to radiation treatment and the commonly used chemotherapeutics [12–14], suggesting that CSCs could be a critical target for cancer therapy.

Tumor microenvironments have limited availability of glucose and the cells undergo competition for nutrients with stromal and immune system cells [15]. Thus, hypoxia and serum depletion are common features of solid tumors that occur during treatment with anti-angiogenesis agents, irradiation and chemotherapy across a wide variety of malignancies [16, 17]. However, the response of tumor cells to hypoxia and serum depletion and the underlying mechanism that mediates this response remains to be clarified. These microenvironmental and metabolic adaptations of cancer cells play important roles in tumor initiation, progression and metastasis.

Nitric oxide (NO), which is synthesized by a family of enzymes called NO synthases (NOS), is a key signaling molecule that mediates various biological, physiological, and pathological processes, including vasodilation, neurotransmission, host defense and cancer progression [18]. Endogenous NO can modulate mitochondrial function [19] and continuous exposure to moderate-to-high concentrations of NO promotes neoplastic transformation [20]. However, the detailed molecular mechanisms by which NO regulates mitochondrial function and tumorigenesis in cancer cells remain incompletely understood.

The expression of specific oncoproteins, such as HRAS, promotes tumor survival and proliferation. Several studies have shown that oncogenic HRASG12V signaling promotes mitochondrial dysfunction and subsequent metabolic reprogramming to favor increased glycolytic flux and glutaminolysis [21, 22]. However, the mechanisms by which HRAS induces mitochondrial dysfunction and its effects on energy metabolism are poorly understood. The adenosine monophosphate (AMP)-activated protein kinase (AMPK), a critical energy sensor of cellular energy homeostasis, is involved in multiple signaling networks to coordinate a wide array of compensatory, protective and energy-sparing responses [23]. NO interacts with AMPK and induces mitochondrial biogenesis [24] and therefore NO and AMPK might be involved in tumorigenesis in many cancer cells.

In this study, we first established *HRasG12V* transfected mouse embryo fibroblast (MEF) cells and investigated the phenotype of cancer mitochondria. Then, we demonstrated how serum depletion affects mitochondria functions, NO synthesis, CSC features and tumorigenesis. Then, we investigated whether the anti-diabetes drug metformin and the NOS inhibitor SEITU suppress mitochondrial OCR and tumorigenesis.

## RESULTS

### HRASG12V transiently suppresses mitochondrial respiration

**Figure 1:**
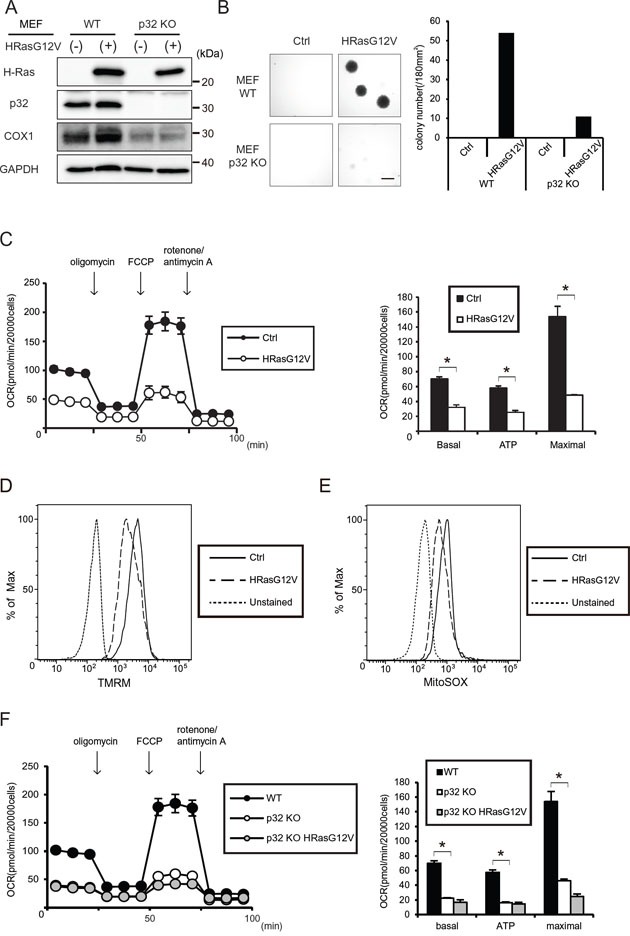
Tumorigenesis and mitochondrial respiratory function of HRASG12V-expressing wild type (WT) and p32 knockout (KO) MEF cells **A.** Immunoblotting analysis of p32 (a mitochondrial RNA chaperone protein) and COX1 (a mitochondrial respiratory complex subunit) expression. **B.** Soft agar assay of WT or p32 KO MEF cells transfected with the control (Ctrl) vector or *HRasG12V*. After 2 weeks incubation, colonies larger than 20,000 μm^2^ per 180 mm^2^ were counted. The microscopic image and histogram show the colony numbers of each sample. Scale bar = 1 mm. **C.** Oxygen consumption ratio (OCR) of control and *HRasG12V* expressing MEF cells. OCR was measured by using an XFe24 analyzer. The histogram shows the basal respiration rate (Basal), ATP production rate (ATP) and maximal respiration rate (Maximal) calculated from the left line chart. Data show the mean ± SD of quadruplicate assays and *p < 0.05; control versus *HRasG12V*. **D.** Mitochondrial membrane potentials (MMP) using a TMRM probe were measured in HRASG12V-expressing MEF cells by FACS analysis. Unstained cells were used as a negative control. **E.** ROS production was measured in HRASG12V-expressing cells by using a MitoSOX probe and FACS analysis. **F.** OCR of WT MEF cell, control and *HRasG12V*-transfected p32 KO MEF cells. *p < 0.05; WT control versus p32 knockout control.

To evaluate the potential role of oncogenic HRASG12V in the decline in mitochondrial respiratory chain activity as a metabolic symptom of the Warburg effect, we established an HRASG12V-expressing cell line to investigate whether HRASG12V expression might alter mitochondrial function. Retroviral vectors expressing wild type (WT) HRAS or oncogenic HRASG12V were used to transform mouse embryo fibroblast (MEF) cells (Figure 1A and Supplementary Figure S1A). The expression of HRASG12V led to a dramatic change in cell morphology characterized by a condensed nuclei and small cell size (Supplementary Figure S1B).

MEF cells expressing WT HRAS or the HRASG12V mutant both exhibited the ability to form colonies in soft agar under 10% serum conditions, whereas only the HRASG12V-expressing cells formed colonies under 1.5% serum conditions in an anchorage-independent colony formation assay (Figure 1B and Supplementary Figure S1C). This shows that HRASG12V-expressing cells have the ability to form colonies in a low serum environment.

Next, we measured the mitochondrial function via OCR in HRASG12V-expressing MEF cells using a Seahorse Flux analyzer. As shown in Figure 1C, an approximately 50% decrease in mitochondrial respiration, including the basal respiratory capacity, the OCR-linked maximal respiratory capacity and the calculated ATP turnover value was detected in HRASG12V-expressing MEF cells. To further investigate the molecular basis for the HRASG12V-induced mitochondrial OXPHOS suppression, we measured the mitochondrial membrane potential (MMP) and ROS by using specific dyes, TMRM and MitoSOX, respectively. Expression of HRASG12V caused a clear decrease in the MMP and ROS production (Figure 1D and 1E). These results suggest that *HRasG12V* transformation leads to suppression of mitochondrial OXPHOS function.

### Mitochondria OXPHOS are necessary for tumorigenesis

The mitochondrial electron transport chain is required for tumor initiation, growth and metastasis [25]. To evaluate the need for mitochondrial OXPHOS during *HRasG12V*-induced transformation, we use p32 knock out (KO) MEF cells, which have OXPHOS dysfunction because of mitochondrial translation inhibition [26]. The p32 KO MEF cells showed reduced mitochondrial encoded COXI expression and a lower respiratory capacity (Figure 1A and 1F). As shown in Figure 1B and 1F, when HRASG12V was expressed in p32 KO MEF cells, they exhibited little ability to form colonies in soft agar while they showed a reduced maximal respiratory capacity similar to that of the HRASG12V-expressing WT MEF cells. The inability of p32 KO MEF cells expressing HRASG12V to grow in an anchorage-independent manner suggests that mitochondrial OXPHOS is crucial during the *HRasG12V*-induced transformation process because the parental p32 KO MEF cells intrinsically have a defect in OXPHOS in contrast to the parental WT MEF cells.

### Serum depletion recovered mitochondrial OXPHOS

**Figure 2:**
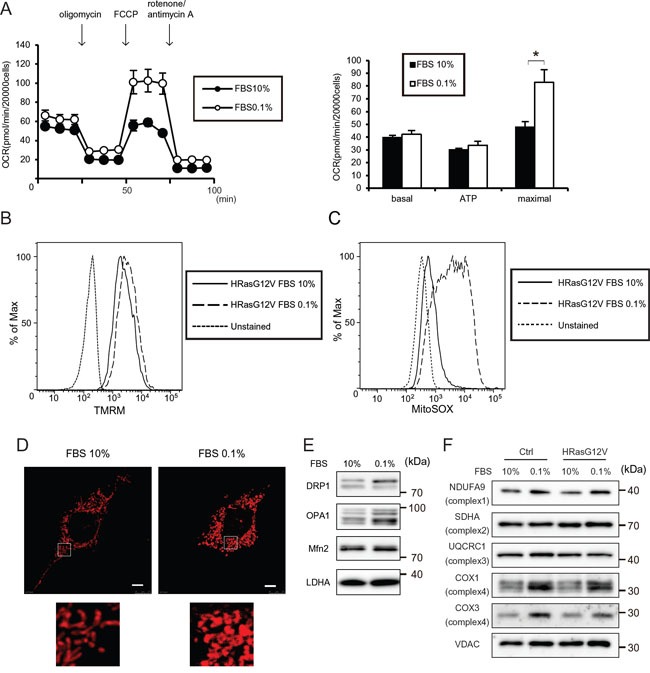
Serum depletion increases the mitochondrial respiratory function of HRASG12V-expressing MEF cells **A.** OCRs were measured after serum depletion (0.1% FBS) for 12 hr. Data show the mean ± SD of quadruplicate assays and *p < 0.05; 10% FBS versus 0.1% FBS. **B.** MMP were measured after serum depletion for 12 hr of HRASG12V-expressing MEF cells by FACS analysis. **C.** ROS production was measured after serum depletion for 12 hr in HRASG12V-expressing MEF cells by using a MitoSOX probe and FACS analysis. **D.** MitoTracker Red staining of HRASG12V-expressing MEF cells incubated with different serum concentrations. Scale bar = 5 μm. The lower image is the enlarged region denoted in the upper image. **E** and **F.** Immunoblotting analysis of mitochondrial fission/fusion proteins (E) and mitochondrial respiratory subunit proteins (F) of HRASG12V-expressing MEF cells. Cells incubated with each medium for 12 hr were subjected to SDS-PAGE.

Serum depletion and hypoxia are common features of solid tumors that occur during anti-angiogenesis, irradiation and chemotherapy across a wide variety of malignancies [16, 17]. To investigate whether serum depletion affects mitochondrial function or morphology, we measured the OCR, MMP and ROS activity after serum depletion in HRASG12V-expressing MEF cells. Serum depletion in HRASG12V-expressing MEF cells increased the OCR-linked maximal respiratory capacity (Figure 2A), MMP (Figure 2B) and ROS production (Figure 2C), indicating that mitochondrial OXPHOS activity recovered after serum depletion in HRASG12V-expressing MEF cells. These results suggest that high concentrations of serum inhibit OCR. We next investigated whether some growth factors such as EGF inhibit OCR. We measured MMP in the presence of EGF with 0.1% FBS or dialyzed 10% FBS. Dialyzed FBS and EGF did not affect the MMP, suggesting that low molecular weight substances such as growth factors did not suppress OCR (Supplemental Figure S2).

Staining cells with MitoTracker Red revealed aberrant dot-shaped mitochondria lacking the normal network connection after serum depletion (Figure 2D), indicating that serum depletion affected the mitochondrial OXPHOS and morphology in HRASG12V-expressing MEF cells, and suggesting that the activity of mitofusion or mitofission was affected by serum depletion. We observed increases in DRP1 and expression of the short form of OPA1 after serum depletion (Figure 2E), suggesting that mitochondria fission activity was increased after serum depletion, leading to morphological changes of mitochondria.

We investigated whether the increased mitochondrial respiration occurred via enhanced gene expression or translation of mtDNA-encoded oxidative phosphorylation complex subunits. No increase of mtRNA was observed, suggesting that the mitochondrial alterations induced by serum depletion were not caused by alterations of mitochondrial genetic materials (Supplementary Figure S3). We did observe increased levels of COXI and COXIII proteins, which are encoded by mitochondrial DNA, and slightly increased levels of the NDUFA9 protein, which is involved in the respiratory complex (Figure 2F), suggesting that serum depletion affects mitochondrial translation and the increased respiration is partially caused by increased levels of respiratory complex protein.

### Serum depletion induces a CSC phenotype in HRASG12V-expressing cells

**Figure 3:**
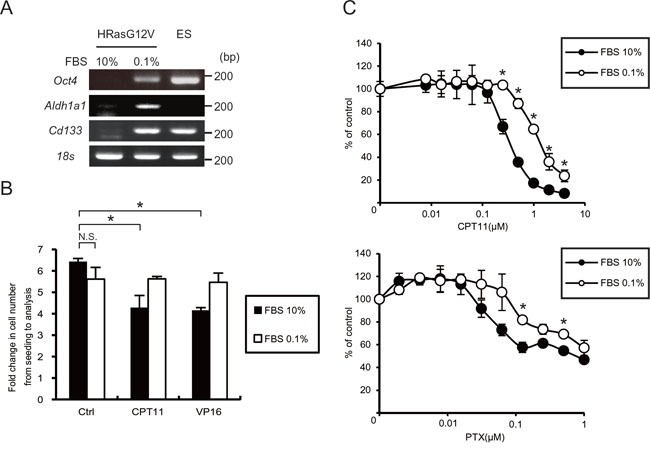
Serum depletion induced CSC gene expression and resistance to anti-cancer drugs **A.** *Oct4, Aldh1a1* and *Cd133* gene expression were measured by RT-PCR analysis after serum depletion of HRASG12V-expressing MEF cells for 48 hr. Embryonic stem (ES) cells were used as a positive control. 18s rRNA was used as a loading control. **B.** Cell proliferation assay of HRASG12V-expressing MEF cells. Cells were treated with CPT11 (0.05μM) or VP16 (0.1μg/ml) with different FBS concentrations and durations (10% for 36 hr or 0.1% for 72 hr). The fold change in cell numbers was used as a measure of proliferation. Data show the mean ± SD of quadruplicate assays. **C.** MTS assay; cells treated with serum depletion for 24 hr were treated with various concentrations of CPT11 and Paclitaxel(PTX) for another 48 hr. Data were normalized to the control. *p < 0.05; 10% FBS versus 0.1%.

Tumor cells expressing CD133, a marker for colorectal cancer initiating or stem cells, survive and are consequently enriched under serum depletion conditions [27]. We investigated whether hypo-nutrients could induce the CSC phenotype in *HRasG12V*-transformed cells. Using polymerase chain reaction (PCR) analysis we observed that the expression of CSC markers such as *Oct4*, *Cd133* and *Aldh1a1* were increased after serum depletion (Figure 3A). Because CSCs are relatively resistant to commonly used chemotherapeutics, we next investigated their chemoresistance. Irinotecan (CPT11) and etoposide (VP16), commonly used drugs for testing the viability of cancer cells, inhibited the cell growth of HRASG12V-expressing MEF cells at the normal serum concentration; the inhibitory effects were barely evident after serum depletion (Figure 3B). As shown in Figure 3C, we observed chemoresistance against CPT11 and paclitaxel (PTX) by an MTS assay, suggesting that serum depletion causes *HRasG12V*-transformed cells to exhibit a CSC-like phenotype and develop chemoresistance.

### Serum depletion induced NO synthesis in *HRas* transformed cells

**Figure 4:**
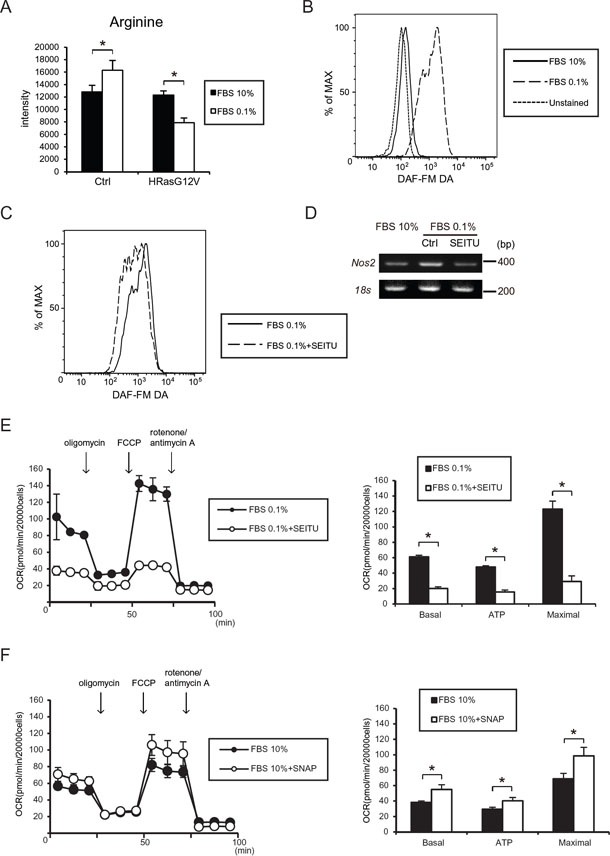
Serum depletion induced NO synthesis and *iNos (Nos2)* gene expression **A.** Intracellular arginine level was measured by liquid chromatography coupled with tandem mass spectrometry (LC-MS/MS). Data show the mean ± SD of quadruplicate assays and *p < 0.05; 10% FBS versus 0.1%. **B, C.** FACS analysis shows intracellular NO production by using the DAF-FM DA probe in HRASG12V-expressing MEF cells after serum depletion for 30 min (B) or pretreated with the NO inhibitor SEITU (C) for 3 hr and then were subjected to serum depletion (0.1% FBS) for another 30 min. **D.** RT-PCR analysis of *iNos* (*Nos2*) gene expression in HRASG12V-expressing MEF cells incubated with serum depletion for 48 hr or pretreated with SEITU (250 μM) for 12 hr before serum depletion. 18s rRNA was used as a loading control. **E.** Cells pretreated with SEITU for 6 hr were incubated with 0.1% FBS for 12 hr. OCR were measured by using an XFe24 analyzer. Data show the mean ± SD of quadruplicate assays and *p < 0.05; 0.1% FBS vs 0.1% FBS + SEITU. **F.** OCR of HRASG12V-expressing MEF cells incubated with or without the NO donor S-nitroso-N-acetylpenicillamine (SNAP, 2 μM). SNAP was added 2 hr before the assay started under 10% serum conditions. *p< 0.05; 10% FBS vs 10% FBS + SNAP.

Because serum depletion affects the contents of amino acids in tumor cells, we measured by LC/MS the amino acid contents in HRASG12V-expressing cells after serum depletion. L-arginine was decreased in HRASG12V-expressing cells after serum depletion (Figure 4A). L-arginine is also a substrate for NO synthesis by nitric oxide synthases (NOS) and NO generation may promote tumor progression. Thus, we investigated NO synthesis after serum depletion in HRASG12V-expressing MEF cells by using a DAF-FM diacetate probe. Fluorescence activated cell sorting (FACS) and immunofluorescence analysis showed significantly increased NO synthesis after serum depletion (Figure 4B and Supplementary Figure S4A). We also found that increased NO production was correlated with a decreased serum concentration, suggesting that serum depletion induced NO synthesis (Supplementary Figure S4B).

Tyrosine nitration is becoming increasingly recognized as a prevalent, functionally significant post-translational protein modification that serves as an indicator of NO-mediated oxidative reactions [28]. We found increased protein nitration in HRASG12V-expressing MEF cells after serum depletion (Supplementary Figure S4C).

Pretreatment with SEITU, which is a non-selective NOS inhibitor, inhibited NO synthesis as shown by FACS analysis (Figure 4C). We also observed increased *iNos* (*Nos2*) expression after serum depletion and SEITU inhibited this increase (Figure 4D), suggesting that an increase in NO synthesis by iNOS in HRASG12V-expressing MEF cells might be involved in the CSC phenotype and tumorigenesis after serum depletion. We also observed that the NOS inhibitor, SEITU, increased intracellular L-arginine content, suggesting that decreased L-arginine content might be involved in iNOS activation (Supplemental Figure S5).

To investigate whether NO induced by serum depletion is involved in upregulation of OXPHOS, we measured the OCR after pretreating with SEITU. SEITU suppressed the OCR in HRASG12V-expressing cells (Figure 4E), suggesting that NO production is involved in OCR after serum depletion. Next, to investigate whether the NO donor S-nitroso-N-penicillamine (SNAP) directly increased the OCR under normal serum conditions, we measured the OCR after 2 μM of SNAP treatment under 10% serum conditions. As shown in Figure 4F, the low dose NO donor SNAP induced an increase in the OCR, suggesting that NO production after serum depletion is directly involved in OCR activity in HRASG12V-expressing cells.

### *HRas* transformation and serum depletion: signal transduction

**Figure 5:**
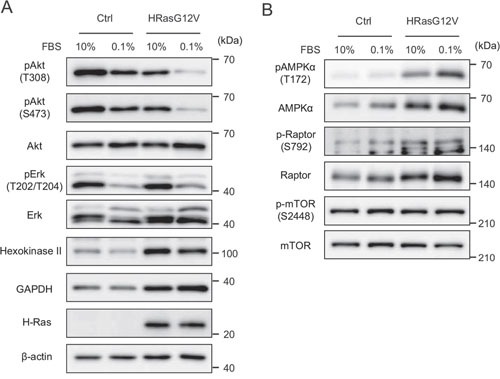
Signal transduction of wild type HRAS and HRASG12V-expressing MEF cells under serum-depleted conditions Cells were incubated with different serum concentrations for 24 hr. Immunoblotting analysis of **A.** RAS pathway and glycolysis **B.** AMPK and mTOR pathway proteins.

Activated HRAS signals primarily through stimulation of the MAPK, PI3K and AKT pathways, and mitochondrial OXPHOS suppression might lead to a change in AMPK and mammalian target of rapamycin (mTOR) pathways. In HRASG12V-expressing cells, phosphorylation of AKT and ERK was slightly decreased and phosphorylation of AKT and ERK clearly decreased after serum depletion, suggesting that the AKT and ERK pathways are not involved in *HRasG12V*-induced transformation even under low serum conditions (Figure 5A).

On the other hand, AMPKα and Raptor were increased in HRASG12V-expressing cells, and serum depletion increased the phosphorylation of AMPKα (Figure 5B). These results suggested that increased AMPKα phosphorylation might be involved in tumorigenesis. Next, we analyzed the expression of hexokinase II (HK II) and GAPDH, key enzymes in glycolysis and downstream targets of the AMPK axis. As expected, HK II and GAPDH protein expression were enhanced (Figure 5A). These results suggest that glycolysis and the AMPK axis are upregulated in *HRasG12V*-transformed cells.

### NOS inhibitor SEITU inhibits anchorage independent growth and promotes apoptosis

**Figure 6:**
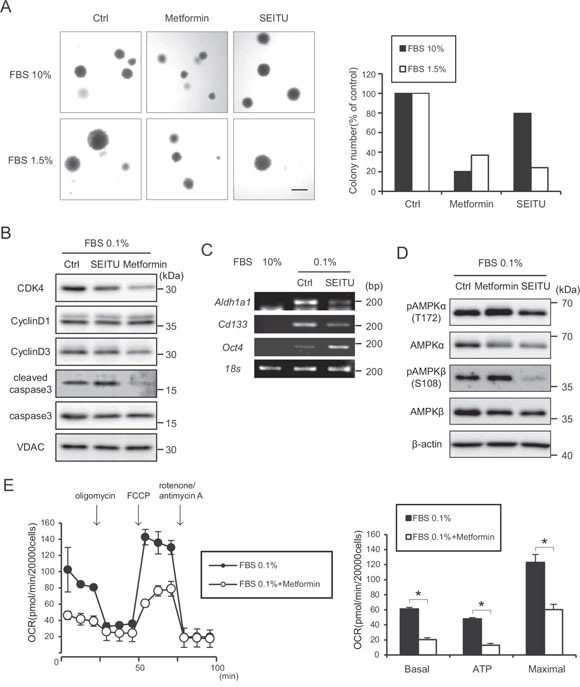
Suppression of anchorage-independent growth effect of SEITU and metformin on HRASG12V-expressing MEF cells **A.** Soft agar colony formation assay of HRASG12V-expressing MEF cells treated with metformin (1 mM), SEITU (250 μM) or not were performed under 10% or 1.5% serum conditions. Left panel is the microscopic image. Scale bar = 1 mm. The right histogram shows colony numbers of each sample. **B.** Immunoblotting analysis: HRASG12V-expressing MEF cells pretreated with SEITU (250 μM) or metformin (1 mM) for 12 hr were serum depleted for another 24 hr. **C.** RT-PCR analysis of gene expression level of cancer stem cell genes in HRASG12V-expressing MEF cells. Total RNA was isolated from HRASG12V-expressing MEF cells incubated with 10% or 0.1% FBS for 48 hr. SEITU (250 μM) was added 12 hr before serum depletion. **D.** Cells pretreated with SEITU (250 μM) or metformin (1 mM) for 12 hr were incubated under serum depletion for another 24 hr. **E.** OCR of HRASG12V-expressing cells treated with or without metformin (1 mM). Metformin was added 6 hr before serum depletion. The serum concentration was 0.1%. Data show the mean ± SD of quadruplicate assays and *p < 0.05; 0.1% FBS versus 0.1% FBS + metformin.

NO generation may promote tumor progression [29]. To determine whether the NOS inhibitor SEITU inhibits anchorage-independent growth, we performed a soft-agar colony formation assay under 10% and 1.5% serum conditions. The SEITU treatment significantly decreased the colony formation ability of HRASG12V-expressing cells under low serum conditions (Figure 6A). Another NOS inhibitor, L-NAME, also inhibited the colony-forming activity of HRASG12V-expressing MEF cells (Supplementary Figure S6). The serum depletion increased the levels of cleaved caspase 3 (Figure 6B). This increase was further augmented by SEITU (Figure 6B), suggesting that serum depletion-induced NO suppresses apoptosis, which is consistent with NOS inhibitors inducing apoptosis in MEF RasG12V expressing cells. SEITU also inhibited the expression of CSC markers *Aldh1a1* and *Cd133* in HRASG12V-expressing cells (Figure 6C). These results clearly demonstrate that serum depletion-induced NO enhances tumorigenesis and the NOS inhibitor SEITU inhibits tumorigenesis by causing apoptosis. We also observed SEITU inhibits the phosphorylation of AMPKα and AMPKβ (Figure 6D), suggesting that increased AMPK phosphorylation after serum depletion is also involved in NO-induced tumorigenesis.

### Metformin inhibits anchorage independent growth by arresting the cell cycle

Metformin is widely used in the treatment of diabetes, and it has been reported that metformin directly acts on mitochondria to limit respiration and may have antineoplastic activity. Metformin decreased the serum depletion-enhanced mitochondrial respiration in HRASG12V-expressing MEF cells (Figure 6E). Metformin also inhibited the colony forming activity of HRASG12V-expressing MEF cells in both the 10% and 0.1% serum conditions (Figure 6A). Metformin did not induce apoptosis but did reduce cyclin D3 and Cdk4 protein expression (Figure 6B), which may arrest the cell cycle in G0/G1. These results suggest that metformin affects the expression of key proteins of the cell cycle and causes HRASG12V-expressing cells to undergo cell cycle arrest.

## DISCUSSION

**Figure 7:**
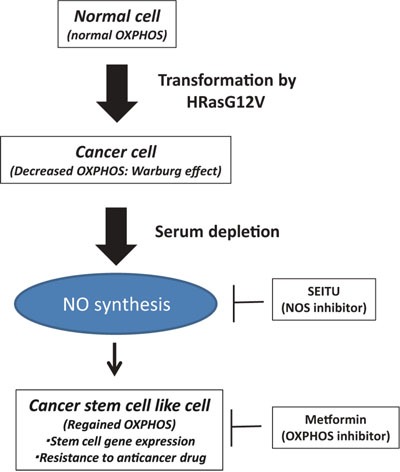
Serum depletion induced NO synthesis and CSC features Oncogenic *HRas* led to suppression of the mitochondrial oxygen consumption rate (OCR) (Warburg effect). In the oncogenic *HRas* transformed cells, serum depletion reversibly increased the OCR, NO synthesis and CSC features. Metformin (anti-diabetic drug) and SEITU (NOS inhibitor) suppress OXPHOS, stem cell gene expression and tumor progression.

The present study showed that serum depletion induces a CSC phenotype because of NO synthesis in oncogenic *HRas*-transformed cells. The major new findings of this study are as follows. (i) Serum depletion reversibly increased the OCR and MMP (Figure 2). (ii) Serum depletion also promoted a CSC phenotype (Figure 3) and NO synthesis (Figure 4B). (iii) SEITU, a NOS inhibitor, inhibited mitochondrial OCR (Figure 4E) and stem cell gene expression (Figure 6C), and reduced anchorage-independent growth by inducing apoptosis (Figure 6A). Our results clearly indicate that serum depletion induces NO production and promotes mitochondrial OXPHOS, leading to tumor progression and a CSC phenotype (Figure 7). In this study, we described the phenotype as CSC like because we did not perform a functional xenograft assay to confirm they were CSCs.

In cancer cells, increased aerobic glycolysis is known as the Warburg effect [3], which has been observed in a variety of malignant tumors. This metabolic change is considered to be a characteristic biochemical symptom of cancer cells. Though Warburg speculated that mitochondrial respiration was injured in cancer cells [3], mitochondrial activity is essential for cancer cell survival. Moreover, mitochondria are involved in hypoxic adaptation regulated by hypoxia-inducible factor 1α, a process that must be triggered in any solid neoplasia to complete the initial steps of tumorigenesis [30].

In the current study, we established wild type *HRas* and *HRasG12V* MEF cell lines to examine the detailed mitochondrial oxygen consumption and metabolic changes that may be associated with tumorigenesis. We also found that the CSC phenotype manifested after serum depletion of genetically normal MEF cells. Oncogenic transformation by *HRas* can lead to mitochondrial dysfunction [31]. Our study showed that expression of oncogenic HRAS protein resulted in a significant decrease in mitochondrial respiration (Figure 1C), accompanied by a decrease of mitochondrial transmembrane potential and ROS generation (Figure 1D and 1E). However, the respiration of the mitochondria recovered after serum depletion (Figure 2A-2C), suggesting that mitochondria reversibly adapt to micro-environmental circumstances.

Oncogenic *KRas* mutations induce CSC of colorectal cancer cells carrying an *Apc* mutation, as shown by comparisons of sphere formation, transforming potential, chemoresistance and expression of stem cell markers [32]. Initial activation of β-catenin by *Apc* loss and further enhancement through *KRas* mutations induces CD44, CD133 and CD166 expression [33]. We speculated that oncogenic *Ras* and another signaling pathway that is activated by hypo-nutrients might induce the CSC phenotype, resulting in resistance to anticancer therapy.

NO is an important mediator of numerous physiological functions, but continuous exposure to moderate-to-high concentrations of NO produced by iNOS may contribute to pathological processes, such as inflammation-associated tissue injury and tumor initiation [20]. Many studies have also indicated that NO can initiate and/or promote tumorigenesis [34]. Conversely, high dose NO promotes DNA damage, gene mutations and tumor cell death, which results in tumor regression and inhibition of metastasis. Oncogenic HRAS also activates iNOS to maintain tumor growth [35].

These characteristics of NO have been exploited therapeutically with impressive effects in pre-clinical models of cancer to slow tumor growth and to enhance the efficacy of chemotherapy. Our results suggest that NO induction by serum depletion is an essential factor for CSC formation and tumor initiation. NOS inhibitors such as SEITU and L-NAME could be useful chemotherapy drugs to inhibit tumorigenesis and the CSC phenotype.

NO induces mitochondrial biogenesis in skeletal muscle cells and NO and AMPK cooperatively regulate PGC1α in skeletal muscle cells. NO interacts with the metabolic sensor enzyme, AMPK [24]. It has been reported that L6 myotubes treated with NO donors, either SNAP or diethylenetriamine-NONO, exhibit elevated AMPK phosphorylation, *Pgc1α* mRNA and protein, and basal and uncoupled mitochondrial respiration [24]. Furthermore, this study supports a proposed model of synergistic interaction between AMPK and iNOS that is critical for maintenance of metabolic function in skeletal muscle cells. In our experiment, oncogenic *HRas* and serum depletion induces AMPK expression, activates its phosphorylation, and is partially involved in tumorigenesis. In addition, the NOS inhibitor SEITU inhibits AMPK phosphorylation (Figure 6D) and tumorigenesis, suggesting that NO and AMPK are essential for tumorigenesis in the hypo-nutrient environment.

Metformin, which is a widely used antidiabetic agent, also suppressed OCR and anchorage-independent growth by inhibiting the cell cycle, suggesting that OCR is one of the targets of metformin. Mechanistically, metformin indirectly activates AMPK signaling and subsequently inhibits mTOR activity, which is frequently increased in cancer cells. In this study, we observed that metformin inhibited OCR and induced AMPK signaling and cell cycle arrest, leading to a reduction in cancer cell proliferation (Figure 6A and 6E).

Once activated, AMPK phosphorylates many downstream effectors to reduce the ATP consuming processes and promote ATP-producing processes [36]. Thus, the observed transient activation of AMPK might promote initial survival. Our results suggest that AMPK is an important component in the overall response to NO; early transient activation promotes recovery and suppresses apoptosis, whereas NOS inhibitors can promote apoptosis. Additional studies are needed to determine the role of AMPK in tumorigenesis and adaptation to insults beyond NO.

Our data provide a molecular mechanism that may contribute to the ability of metformin to prevent or treat cancer. Somehow, metformin suppresses respiration and citric acid cycle activity even in isolated mitochondria, indicating that mitochondria are one of its direct targets [37]. We speculate that metformin accumulates within mitochondria and suppresses mitochondrial respiration, leading to an arrest of cell proliferation. Our and others' observations suggest that metformin can act in cooperation with chemotherapeutic drugs that increase genotoxic stress.

Taken together, our findings are the first to indicate that NO plays a vital role in mitochondrial homeostasis and tumorigenesis after serum depletion in oncogenic *HRas*-transformed cells.

## MATERIALS AND METHODS

### Cell culture

Mouse embryonic fibroblast (MEF) were isolated from WT and p32 knockout C57BL/6 mice as described previously [26]. WT and p32KO MEF cells were used at low passages and were cultured with Dulbecco's modified Eagle's medium (DMEM; Sigma Aldrich) supplemented with 10% fetal bovine serum (FBS) at 37°C in a humidified atmosphere with 5% CO_2_. WT *HRas* and *HRasG12V* genes in a pMXs retroviral vector (Cell Biolabs, Inc.) were transfected into these MEF cells and we established stably expressing cell lines.

### Seahorse XF24 flux analyzer

The oxygen consumption ratio was measured by using an XFe24 Analyzer (Seahorse Biosciences). The cells (2 × 10^4^/well) were seeded into Seahorse XF microplates and incubated at 37°C for 24 hr. The mitochondrial inhibitors were added sequentially: 0.5 μM of oligomycin, 0.75–2 μM of carbonyl cyanide-4-(trifluoromethoxy) phenylhydrazone (FCCP), 1 μM of rotenone and 1 μM of antimycin A. The assay was performed according to the manufacturer's instructions. After the assay was finished, the cells were counted to normalize the values.

### Immunoblotting analysis

Briefly, cells were lysed with lysis buffer (50 mM Tris–HCl, pH 7.5, 1 mM EDTA, 150 mM NaCl and 0.5% NP-40) and then subjected to immunoblotting as described previously [38].

### Measurement of cellular nitric oxide by fluorescence activated cell sorting

Intracellular NO was measured using DAF FM-DA (Sekisui Medical). HRASG12V-expressing MEF cells were treated with 0.1% FBS 15 min before DAF FM-DA staining. The cells were stained with DAF FM-DA (5 μM, 37°C, 15 min) on the dish. The cells were then washed with Hank's balanced salt solution (HBSS) and analyzed by FACS on a BD FACSVerse (BD Biosciences).

### Measurement of mitochondrial membrane potential and ROS by FACS

Mitochondrial membrane potential was estimated using a tetramethyl-rhodamine-methylester (TMRM) probe (ImmunoChemistry Technology). Intracellular ROS were detected using a MitoSOX Red probe (Invitrogen). Cells were seeded on a 6-well dish and incubated at 37°C for 24 hr. They were stained with TMRM (100 nM, 37°C, 30 min) and MitoSox Red (10 μM, 37°C, 10 min) on the dish and trypsinized. The cells were then washed with HBSS and acquired on a BD FACSVerse (BD Biosciences).

### Soft agar colony formation assays

A bottom layer of 0.5% agarose/10% or 1.5% FBS with DMEM (2×) was plated on a 6-well dish. Cells (2.5 × 10^3^) were suspended in 0.35% agarose/10% or 1.5% FBS with DMEM (2×) and overlaid. The culture medium contained the vehicle, metformin (1 mM), or S-ethyl-isothiourea (SEITU) (250 μM). After 2 weeks, the colonies were stained with 0.005% crystal violet. Microscopic images were obtained using an immunofluorescence microscope (BZ-9000; Keyence). Colonies larger than 20,000 μm^2^ were counted in a 180-mm^2^ area.

### Immunofluorescence imaging

Immunofluorescence was carried out according to established techniques [26]. Cells were incubated with 10% or 0.1% FBS for 12 hr before cells were fixed. MitoTracker Red (Invitrogen, 5 nM) and DAF-FM DA (Sekisui Medical) were added directly to the culture medium and incubated for 30 min. Fluorescence images were obtained using a confocal laser microscope (Leica).

### MTS assay

Briefly, cells (10% FBS: 4 × 10^3^ cells/well, 0.1% FBS: 2 × 10^4^ cells/well) in 100 μl of phenol-red free DMEM containing FBS were seeded in each well of a 96-well plate. After 24 hr of incubation, different concentrations of anticancer drugs CPT11 and PTX were added to each well. After 48 hr, 20 μL of the CellTiter 96 Aqueous One Solution (Promega) was added to each well and incubated for 2 hr and then absorbance was measured at 490 nm.

### mRNA quantification

Total RNA was isolated by using an RNeasy Mini Kit (Qiagen) according to the manufacturer's instructions. Reverse transcription of 1 μg total RNA was performed by using a PrimeScript™ RT Reagent Kit (TAKARA). The CSC genes were detected by quantitative PCR by using the SYBR® *Premix Ex Taq*™ II (TAKARA) with a thermal cycler (StepOne plus; Applied Biosystems). The CSC genes were detected by agarose gel electrophoresis. All primer were represented in supplemental Table S1.

### Liquid chromatography-mass spectrometry

The metabolites were analyzed by liquid chromatography-mass spectrometry (LC-MS) based on reverse phase ion-pair chromatography and hydrophilic interaction chromatography modes coupled with a triple quadrupole mass spectrometer (LCMS-8040; Shimadzu) [39].

## SUPPLEMENTARY FIGURES AND TABLE


