# Supplementary material for: Serum depletion induced cancer stem cell-like phenotype due to nitric oxide synthesis in oncogenic HRas transformed cells
Source: Oncotarget. 2016 Sep 19;7(46):75221–34. doi: 10.18632/oncotarget.12117 (PMC5342736; doi:10.18632/oncotarget.12117)
Supplement: Supplementary file 1 [file oncotarget-07-75221-s001.pdf]

Serum depletion induced cancer stem cell-like phenotype due to nitric oxide synthesis in oncogenic *HRas* transformed cells

SUPPLEMENTARY ANTIBODY

Polyclonal antibodies against mouse p32 and VDAC were raised in our laboratory. The primary antibodies against p-AKT at Thr308 (#2965), p-AKT at Ser473 (#4060), AKT (#4691), p-Erk (#9106), Erk (#4695), p-p70S6 Kinase at Thr389 (#9234), p70S6 Kinase (#2708), p-mTOR (#2971), mTOR (#2983), p-Raptor (#2083), Raptor (#2280), p-eIF2α at Ser51 (#3398), eIF2α (#5324), ATF4 (#11815), p-4EBP1 (Thr37/46) (#2855), p-4EBP1 (Ser65) (#9451), 4EBP1 (#9644), pAMPKα (Thr172)(#2535), AMPKα (#2603), pAMPKβ (#4181), AMPKβ(#4150), IRE1α (#3294), CDK4 (#2906), Cyclin D1 (#2926), Cyclin D3 (#2956), cleaved caspase 3 (#9661), caspase 3 (#9665), Hexokinase 2 (#2867), Mitofusin-2 (#9482), Nitro-Tyrosine(#99691), GAPDH (#2118) were purchased by Cell signaling(Beverly, MA, U.S.A), respectively. Another primary antibodies were used NDUFA9 (#459100 invitrogen), SDHA (#459200 invitrogen), coxI (#459600 invitrogen), ACSL4(FACL4) (sc-48997, Santa Cruz), H-Ras (sc-520, Santa Cruz), UQCRC1(ab110252 abcam), FACL4 (ab137525, abcam), OPA1 (#612606 BD Transduction Laboratories), DRP1 (#611112 BD Transduction Laboratories), α-tubulin (PM054, MBL), β-actin (A5441 Sigma-Aldrich), respectively. Secondary antibodies were used Anti-mouse IgG HRP-linked (#7076 cell signaling), Anti-rabbit IgG HRP-linked (#7074 cell signaling), Alexa Fluor® 488 F(ab')2 Fragment of Goat Anti-Rabbit IgG (H+L) (#A11070 Life Technologies), Alexa Fluor® 594 F(ab')2 Fragment of Rabbit Anti-Goat IgG (H+L) (#A11080 Life Technologies).

SUPPLEMENTARY FIGURES AND TABLE

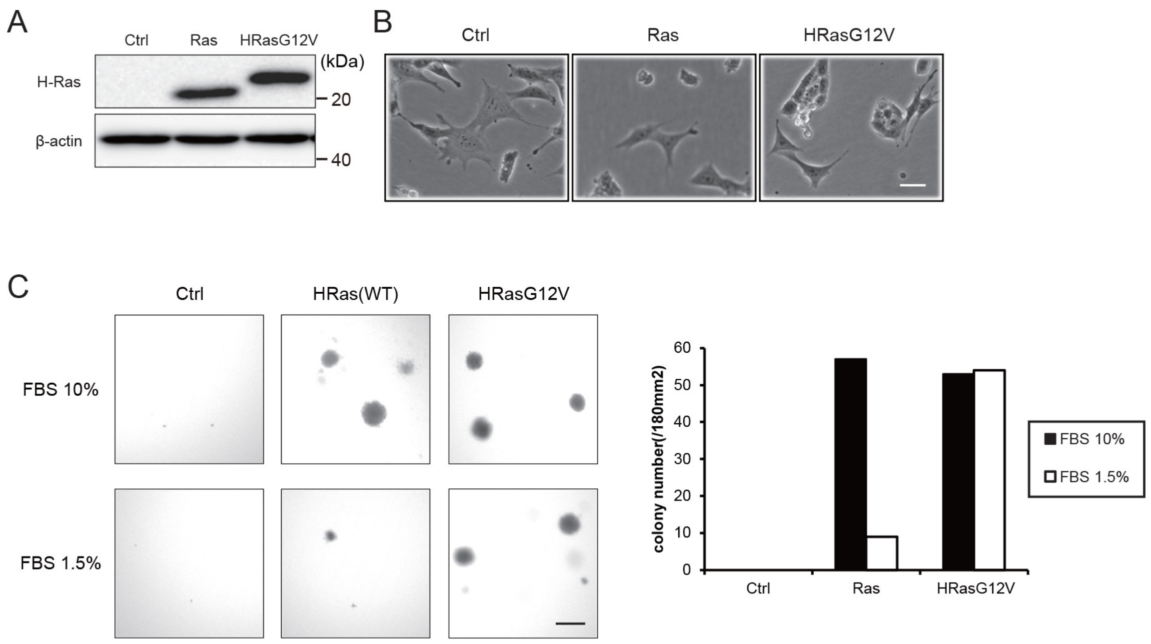

Supplementary Figure S1: Establishment of HRas-expression cells and HRasG12V-expression cells. A. Immunoblotting analysis of Ras protein in each cells. **B.** Bright field microscopic image of MEFs transfected with the control (Ctrl) vector, HRas (Ras) or HRasG12V. **C.** Soft agar assay of each cells. Serum concentration was 10% or 1.5%.

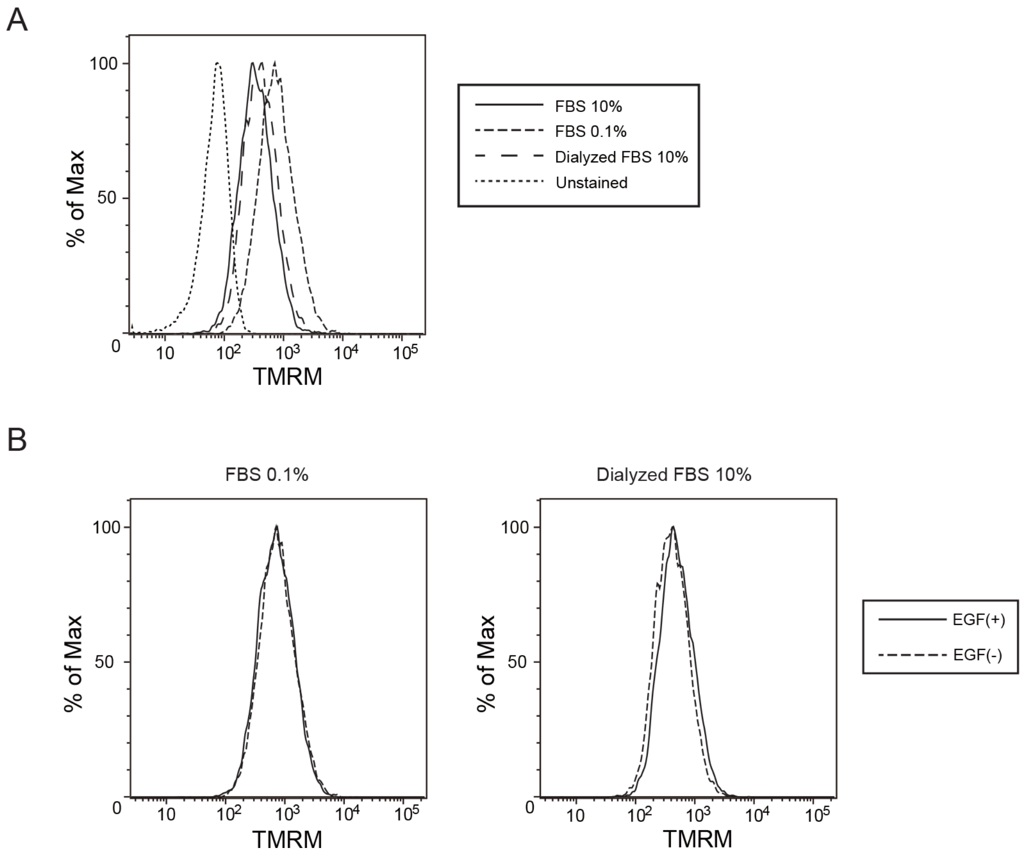

Supplementary Figure S2: MMP were measured after cultured with different condition for 12 hr of HRasG12V-expressing MEF cells by FACS analysis. A. medium contains 10% FBS, 0.1% FBS or 10% dialyzed FBS. **B.** medium contains 0.1% FBS or 10% dialyzed FBS ± EGF(30ng/ml).

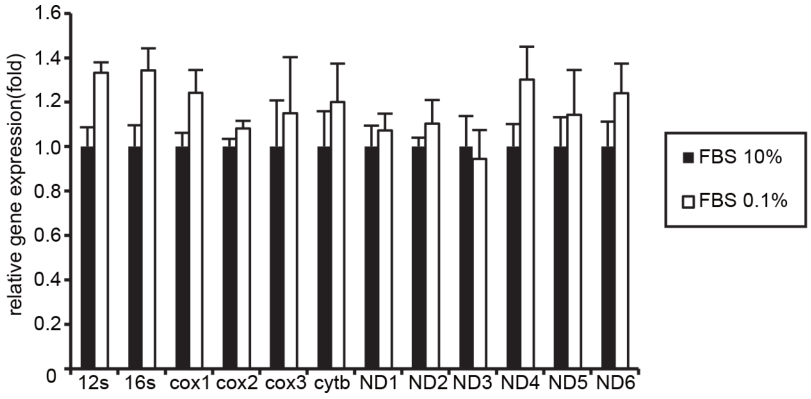

Supplementary Figure S3: Expression of mRNA encoded by mitochondrial DNA under serum depleted condition. Real-time PCR analysis shows mitochondrial gene transcript levels isolated from HRasG12V-expressing cells incubated with different serum concentration. Data were normalized to the expression level in FBS 10% for each RNA species. Data show the mean ± SD of quadruplicate.

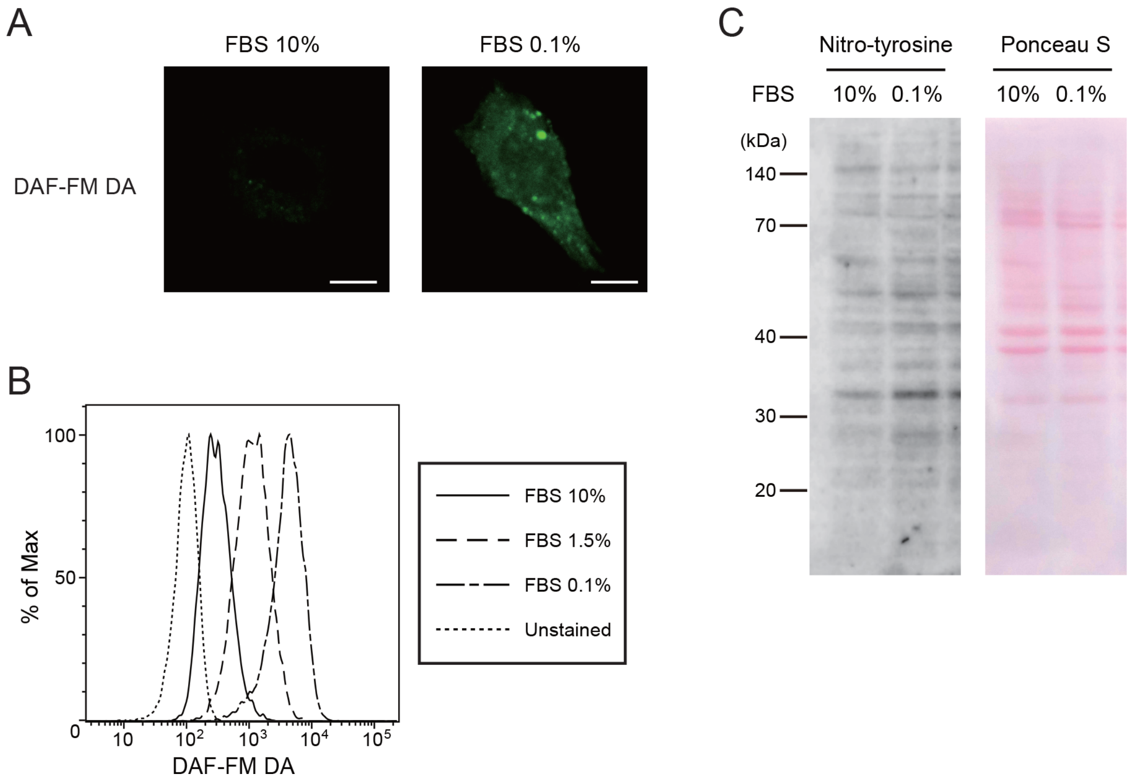

Supplementary Figure S4: Intracellular nitric oxide production under different serum concentration. A. Immunofluorescence staining of DAF-FM DA in HRasG12V-expressing cells incubated with different serum concentrations. Scale bar = 5 µm. **B.** HRasG12V-expression cells which pretreated with SEITU were incubated with different serum concentration (FBS 10%, 1.5% or 0.1%). The intracellular NO production was measured by FACS analysis. **C.** Immunoblotting analysis shows tyrosine nitration by using an anti-nitro tyrosine antibody on HRasG12V expressing cells after serum depletion for 24 hr. The right panel shows ponceau S staining of the same membrane.

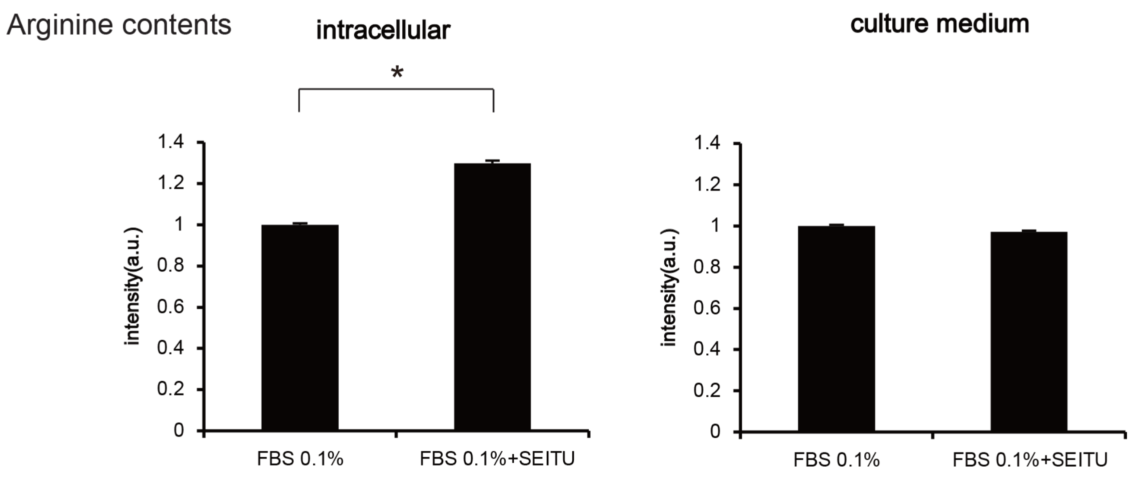

Supplementary Figure S5: L-arginine content in intracellular and culture medium after pretreatment with NOS inhibitor SEITU. L-arginine contents were measured by liquid chromatography coupled with tandem mass spectrometry (LC-MS/MS). Data show the mean ± SD of quadruplicate assays and *$P<0.05$; 10% FBS versus 0.1%.

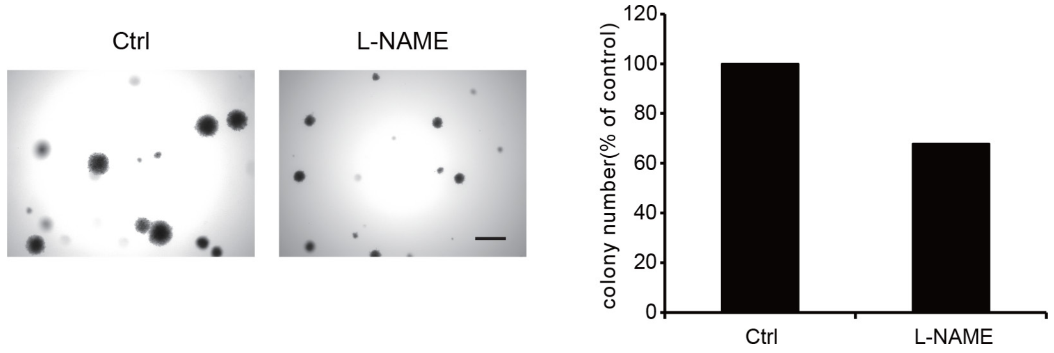

Supplementary Figure S6: Inhibition of anchorage-independent growth by L-NAME. Soft agar assay of HRasG12V-expression cells treated with or without L-N^{G}-Nitroarginine methyl ester (L-NAME, 2.5mM) were performed under 1.5% serum conditions. Left panel is the microscopic image. Scale bar = 1 mm. The right histogram shows colony numbers of each sample.

Supplementary Table S1: Primer sequences of the qRT-PCR primers are as follows

Primer	Forward (5' - 3')	Reverse (5' - 3')
Atf4	tcgatgctctgtttcgaatg	agaatgtaaagggggcaacc
Fgf21	gggaggatggaacagtggta	gtcctccagcagcagttctc
Chop	gcatgaaggagaaggagcag	cttccggagagacagacagg
Gadd34	gacccctccaactctccttc	gcctctaccttggcttctcc
Mthfd2	tgataatcacgagggcagct	acacccgacagatgagcttt
Trib3	gctgtgggattcaagccaaa	ctgtgggcctgggtactaaa
Oct4	aaatcggagaccctggtgca	ttctggcgccggttacagaa
Slc7a5	cacctgccttctgtcctctc	tgaatcggagccacatcata
Aldh1a1	atacttgtcggatttaggaggct	gggcctatcttccaaatgaaca
Cd133	ggacccagaaactggcaaag	tcgccatggccttaatctct
18s rRNA	cgcggttctattttgttggt	agtcggcatcgtttatggtc
Nos2	ctatggccgctttgatgtgc	acctccagtagcatgttggc
12s rRNA	ccgctctacctcaccatctc	cccatttcattggctacacc
16s rRNA	gggataacagcgcaatccta	gattgctccggtctgaactc
Cox1	ggtcaaccaggtgcactttt	tggggctccgattattagtg
Cox2	acgaaatcaacaaccccgta	ggcagaacgactcggttatc
Cox3	caaggccaccacactcctat	attcctgttggaggtcagca
Cytb	tgagggggcttctcagtaga	ctgtttcgtggaggaagagg
Nd1	ggatccgagcatcttatcca	ggtggtactcccgctgtaaa
Nd2	agggatcccactgcacatag	ctcctcatgcccctatgaaa
Nd3	ttcgaccctacaagctctgc	tgaattgctcatggtagtgga
Nd4	ccactgctaattgccctcat	cttcaacatgggcttttggt
Nd5	tcctactggtccgattccac	tttgatgtcgttttgggtga
Nd6	cgatccaccaaaccctaaaa	ttggttgtcttgggttagca
